# The Influence of Storage Conditions on the Quality of Vacuum-Packed Water Caltrop Shell

**DOI:** 10.3390/foods14203567

**Published:** 2025-10-20

**Authors:** Zhihua Wan, Wangping Wang, Xiaopeng Liu, Pengju Li, Wenhao Zhang

**Affiliations:** College of Mechanical Engineering, Wuhan Polytechnic University, Wuhan 430023, China; wwp@whpu.edu.cn (W.W.); lxp1989@whpu.edu.cn (X.L.); 17638640373@163.com (P.L.); 13597942702@163.com (W.Z.)

**Keywords:** water caltrop shell, storage temperatures, sensory quality, texture characteristics, microstructure

## Abstract

In order to explore the influence of storage temperature and time on the quality of vacuum-packed water caltrop shell (WCS), this study investigated the changes in the quality of vacuum-packed WCS under different storage temperature (3 ± 1 °C, 5 ± 1 °C and 7 ± 1 °C) and time. The quality-related parameters of WCS, including sensory quality, moisture content, texture characteristics and microstructure, were examined. The results showed that at the storage temperature of 5 ± 1 °C, vacuum-packaged WCS could maintain high sensory quality within 21 days, while at 3 ± 1 °C and 7 ± 1 °C, the samples showed low sensory quality at 21 days and 14 days, respectively. For the same storage time, storage at 5 ± 1 °C resulted in the least significant decrease in elastic modulus and compressive strength of the samples. Among the three storage temperatures, storage at 7 ± 1 °C led to the most obvious change in pore structure, followed by storage at 3 ± 1 °C and then 5 ± 1 °C. The variance analysis suggested that storage time has significant effects on all the tested parameters, while storage temperature has significant effects on the sensory quality and texture characteristics of the samples but shows no significant effect on the moisture content. These findings provide a theoretical reference for the packaging and storage of WCS and the development of water caltrop sheller.

## 1. Introduction

The water caltrop (*Trapa* spp.), also commonly known as the water chestnut, is an annual herbaceous aquatic plant native to China and has been planted in China for 7000 years. This distinctive vegetable, cultivated for its edible, nut-like fruit encased in a hard, horned shell, holds significant cultural and culinary importance in its region of origin. Renowned for its crisp texture and subtly sweet, nutty flavor, the water caltrop fruit is not only a culinary delicacy but also a valuable source of nutrition. However, the processing and consumption of this fruit generate a substantial by-product: the water caltrop shell (WCS). Research indicates that WCS constitutes approximately 25% of the total mass of the harvested fruit, representing a significant volume of residual biomass [1].

Traditionally, this shell material has been largely undervalued and considered waste. Common disposal practices have included direct discarding, composting, or use as low-grade animal feed. Unfortunately, these methods often lead to environmental concerns, such as contributing to landfill burdens or potentially causing localized pollution if not managed properly, alongside representing a clear inefficiency in resource utilization. This underutilization is particularly regrettable because scientific studies have increasingly revealed that WCS is far from inert waste. It is, in fact, a rich reservoir of diverse bioactive compounds. Analyses confirm the presence of valuable constituents such as polyphenols, polysaccharides, and notably, a significant content of flavonoids [2,3]. These flavonoids, among other components, have demonstrated promising biological activities in preliminary research, including potential anti-cancer effects, suggesting considerable medicinal value [2,3].

Consequently, WCS is attracting growing interest from the pharmaceutical and nutraceutical industries seeking natural bioactive sources, as well as the food industry exploring functional ingredients or natural preservatives. Despite this emerging recognition of its potential and the inherent waste and environmental issues associated with current practices, the widespread commercial exploitation of WCS remains limited. A primary bottleneck hindering efficient utilization is the shell’s inherent perishability. WCS possesses low durability during storage and is highly susceptible to rapid deterioration—likely due to microbial growth, enzymatic activity, or moisture-related spoilage—shortly after harvest and processing [4,5]. This perishability makes collection, transportation, and storage for large-scale processing or extraction economically and logistically challenging. Therefore, developing effective, practical, and economical methods and conditions for preserving water caltrop shells post-harvest is critically important. Overcoming this storage hurdle is fundamental to unlocking the full economic potential of WCS, mitigating environmental impacts from waste, and realizing the benefits of its valuable bioactive compounds for various applications.

Currently, air-conditioned packaging, coating packaging and vacuum packaging are commonly used methods for the packaging of fruits and vegetables [6,7]. Among them, vacuum packaging can prevent the oxidation of fruit and vegetable epidermis and inhibit the growth of microorganisms and bacteria and has the advantages of low cost and high safety [8,9]. However, vacuum packaging can inhibit the respiration of fresh fruit, making it highly hypoxic and even causing physiological diseases [10]. Therefore, vacuum packaging is not widely applied to the packaging of fresh fruit and vegetable products. Nevertheless, vacuum packaging is highly suitable for the packaging of agricultural products with tight epidermal structure and dormancy. Sujeetha et al. [11] reported that vacuum packaging can protect the pomegranate peel from oxidation and deterioration so as to extend its shelf life. In the study of organically grown bananas in Barangon, Elda et al. [12] found that vacuum packaging could maintain good sensory quality of bananas at the ripening stage and reduce the occurrence of banana crown rot. In addition, vacuum packaging has also been widely used in the preservation and storage of lotus seeds, potato, chestnut and other agricultural products [13,14,15]. Nonetheless, there has been no report on the application of vacuum packaging to the storage of WCS.

Temperature is an important factor affecting the storage quality of fruits and vegetables [16,17]. Li et al. [18] explored the influence of storage temperature on the postharvest quality of water caltrop and revealed that low temperature storage could significantly reduce water loss and respiratory intensity of water caltrop. Zubala et al. [19] found that low temperature storage could inhibit the browning of water caltrop and extend its shelf life. At present, research on water caltrop is mainly focused on the analysis of its nutritional components, food processing methods, and medicinal value of WCS [20,21,22].

It is noteworthy that aquatic vegetables, due to their unique growing environment and high moisture content, exhibit significantly different postharvest deterioration mechanisms compared to terrestrial vegetables. Recent years have seen growing research interest in the decay mechanisms of aquatic vegetables under vacuum packaging conditions. A study on starch-based aquatic vegetables (e.g., arrowhead, water bamboo, lotus root, water chestnut) revealed that these vegetables are prone to respiration, transpiration, and enzymatic browning during transportation, marketing, and storage, leading to a series of rapid quality deteriorations such as shrinkage, water loss, texture softening, and browning. The study pointed out that although vacuum packaging can reduce oxygen exposure, biochemical reactions within the vegetables can still persist if enzyme activity is not effectively inhibited [23].

Another study on vacuum-packed tender ginger found that temperature fluctuations significantly accelerate the deterioration process of vacuum-packed products. Under logistics conditions with frequent temperature variations, even with vacuum packaging, total viable bacterial count and total volatile basic nitrogen indicators increased markedly, indicating continued microbial activity and enzymatic reactions [24]. This suggests that when designing a vacuum packaging strategy for WCS, not only temperature control but also temperature stability must be maintained.

Addressing the specific characteristics of aquatic vegetables, researchers have developed combined preservation technologies. For instance, a method for controlling the deterioration of starch-based aquatic vegetables employs combined treatment of electrostatic field processing and chitosan-tea polyphenol-sesame oil Pickering emulsion coating. This approach inactivates peroxidase and polypoxidase activity via the electrostatic field, followed by coating to inhibit transpiration and respiration, thereby synergistically delaying quality deterioration [23]. This provides a new perspective for WCS preservation: vacuum packaging can be integrated with other pre-treatment technologies to form multiple protective barriers.

However, there have been few reports about the effect of storage temperature on the quality and texture characteristics of WCS. In our preliminary experiments, we found that storage temperature has a significant effect on the sensory quality and microstructure of WCS. Therefore, this study examined the parameters of sensory quality, moisture content, texture properties and microstructural characteristics of WCS to evaluate the quality changes in vacuum-packed WCS under different storage temperature, aiming to obtain the optimal storage temperature and time, and provide theoretical reference for the packaging, storage and development of related machinery for WCS.

## 2. Materials and Methods

### 2.1. Materials

**Figure 1:**
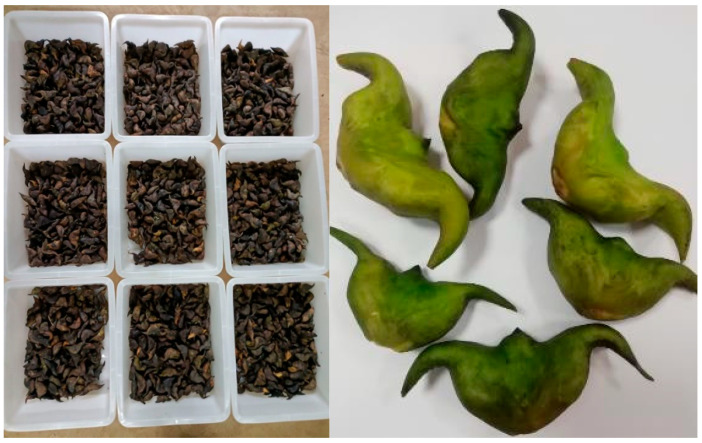
Water caltrop fruit for test.

Plump freshwater caltrop fruits with consistent maturity and no mechanical damage, diseases and insect pests were used as raw materials for the experiment (Figure 1). The samples were collected from Honghu City, Hubei Province, at the end of August 2024.

### 2.2. Main Experimental Instruments and Equipment

The moisture content was measured with the SDH-1202 rapid halogen moisture tester (Zhejiang Said Instrument Equipment Co., Ltd., Hangzhou, China), the texture properties were determined with the TMS-PRO texture instrument (maximum load of 1000 N, detection accuracy of ±1.5%, loading speed of 0.1–500 mm/min, Food Technology Corporation, Sterling, VA, USA), the microstructural characteristics were examined with the SU8010 Scanning Electron Microscope (Toshiba Corporation, Kawasaki-shi, Japan), the vacuum degree was controlled by the Model 600 External Vacuum Packing Machine (Wuhan Xinghua Tengda Machinery Equipment Co., Ltd., Wuhan, China), the storage temperature was controlled by GTTH-S-80 constant temperature and humidity test chamber (Hubei Gaotian Test Equipment Co., Ltd., Wuhan, China).

### 2.3. Test Methods

#### 2.3.1. Sample Treatment

**Figure 2:**
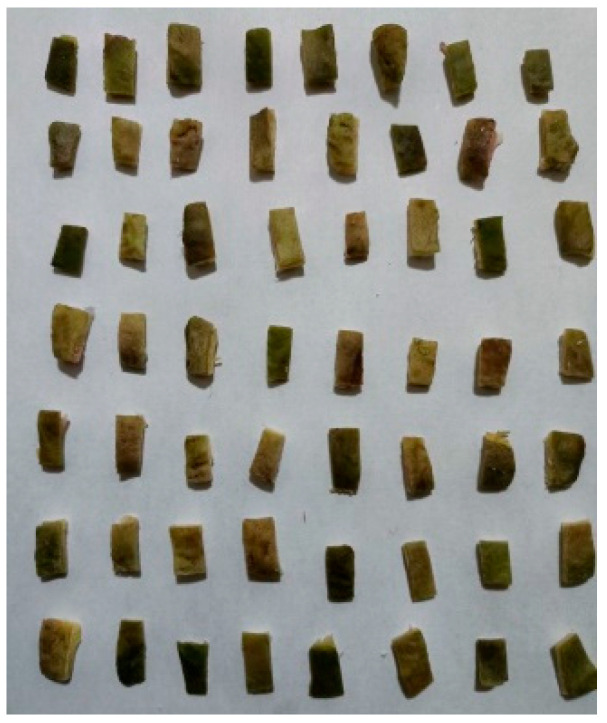
Partial test samples (WCS).

Fresh water caltrop fruit were immediately transported to the laboratory after picking and shelled by hand. The shells were cut into rectangular shape (length of about 8–14 mm and width of about 4–8 mm) to prepare samples as shown in Figure 2. A total of 600 samples were prepared, which were packed into PE film plastic bags and vacuum treated with a 600 external vacuum packing machine. The vacuum pressure in the packing bags was –0.09 MPa [25]. The samples were divided into three groups labeled as A, B and C and then stored in three constant temperatures (3 ± 1 °C, 5 ± 1 °C, 7 ± 1 °C) and constant humidity for 28 d. The sensory quality, moisture content and texture characteristics were measured every 7 d, and the microstructure of the samples was observed before storage and after 28 d [26]. Immediately after each sampling, the remaining samples were repackaged in the original packing manner to ensure that the test environment remained unchanged throughout the test period.

#### 2.3.2. Determination of Sensory Quality

Sensory quality represents the most immediate and perceptible measure for evaluating the post-harvest condition and overall acceptability of WCS. Unlike instrumental analyses that require specialized equipment, sensory attributes-primarily encompassing color, odor, and surface texture-provide a direct, human-readable assessment of quality that correlates strongly with freshness, potential spoilage, and suitability for further processing or extraction of valuable compounds. Changes in these parameters often serve as early, visible indicators of biochemical degradation, microbial activity, or physical damage incurred during handling or storage.

To obtain a robust and reliable assessment of these critical sensory characteristics, a panel of ten experienced experts was assembled. The selection of experienced panelists is crucial to minimize subjective bias and ensure consistency, as they possess trained sensory acuity and a calibrated understanding of the quality benchmarks specific to WCS. These experts conducted evaluations under controlled conditions designed to minimize external influences, likely including standardized lighting (especially important for color assessment) and neutral ambient odors.

The evaluation employed a digital scoring method [27,28], a structured and quantifiable approach that translates subjective sensory perceptions into objective numerical data. This method enhances precision and reproducibility compared to descriptive analyses. Each expert’s sensory attributes (color, odor, surface texture) are independently rated. The overall acceptability score is indeed a separate follow-up rating given by experts based on their overall impression, rather than a mathematical average of individual attributes. Each sensory attribute was systematically assessed and assigned an individual score by each panelist. The scoring system utilized a clearly defined five-level scale.

Score 5: Excellent Quality—Indicating ideal characteristics: vibrant, uniform, typical color; fresh, characteristic, pleasant odor; intact, firm, optimal surface texture.

Score 4: Good Quality—Minor, acceptable deviations from the ideal state.

Score 3: Fair/Acceptable Quality—Noticeable but not severe deterioration; product remains usable, but quality is reduced.

Score 2: Poor Quality—Significant deterioration evident in one or more attributes; generally unacceptable for further use.

Score 1: Very Poor Quality—Pronounced spoilage: severe discoloration (e.g., darkening, mold); strong off-odors (rancid, fermented, putrid); severely degraded texture (slimy, mushy, brittle).

After individual evaluations, the scores given by all ten experts for each sample and each attribute were compiled. To derive a single, representative value for the sensory quality of each sample, the average score across the expert panel was calculated. This average score served as the primary test result for sensory evaluation, providing a consolidated metric reflecting the collective expert judgment on the sample’s overall sensory acceptability and condition. The specific criteria defining each score level for color, odor, and texture were detailed in Table A1, ensuring transparency and consistency in the application of the scale throughout the study.

#### 2.3.3. Determination of Moisture Content

**Figure 3:**
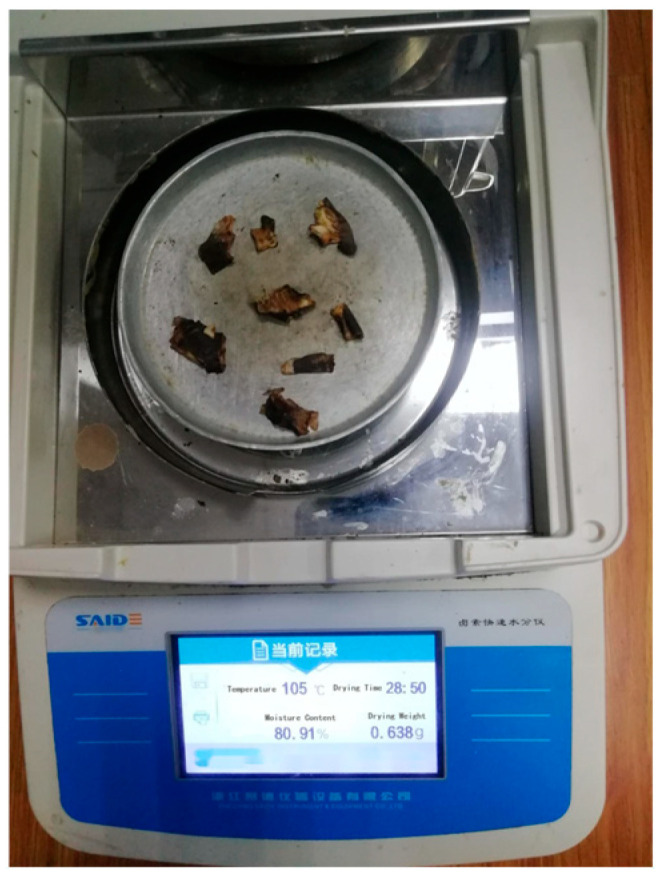
Measurement of moisture content.

Moisture content stands as a critical quality parameter for evaluating WCS, fundamentally linked to its susceptibility to spoilage and degradation. Elevated moisture levels create a favorable environment for microbial proliferation, particularly molds, which can rapidly colonize the shell material, leading to visible deterioration, off-odors, and a significant loss in both nutritional and functional value, as highlighted in prior research [18]. Consequently, precise moisture monitoring is essential for assessing shelf-life and storage stability. To establish a baseline, the initial moisture content of each experimental sample group was meticulously determined before the commencement of the storage trials. Following each designated sampling interval, the moisture content of the retrieved WCS samples was quantitatively assessed using an SDH-1202 rapid halogen moisture analyzer. This instrument operates by rapidly heating the sample with a halogen lamp and precisely measuring the weight loss due to moisture evaporation. To ensure accuracy and reliability, five independent replicate measurements were performed for every sample at each time point. The resulting values were then statistically averaged to yield a single, representative moisture content datum per sample/time point. This comprehensive set of averaged moisture data, charted over the course of the experiment, is graphically presented in Figure 3, illustrating the temporal dynamics of water loss or retention under different storage conditions.

#### 2.3.4. Measurement of Textural Characteristics

Beyond sensory attributes, the textural characteristics of WCS provide crucial internal indices for assessing its structural integrity and overall quality, directly impacting its suitability for processing and potential extraction efficiency. Two key mechanical properties were measured: elastic modulus (quantifying the material’s inherent stiffness or resistance to elastic deformation under stress) and compressive strength (indicating the maximum stress the shell can withstand before failure or rupture) [29]. These parameters offer objective insights into the biomechanical integrity and degradation state of the WCS. Measurement was performed using a TMS-PRO texture analyzer, a precision instrument designed for controlled force/deformation testing. The compression setup consisted of:

An Upper Compression Probe: A rigid, flat-ended cylindrical plate (P/72 probe type) with a diameter of 72 mm and a thickness of 6 mm.

A Lower Support Base: A rectangular steel plate measuring 110 mm (length) × 100 mm (width) × 10 mm (thickness), providing a stable, rigid platform.

To account for potential anisotropy in the shell structure, compression tests were conducted along three mutually perpendicular axes:

Horizontal (H) direction, corresponding to the sample’s height dimension.

Lateral (L) direction, corresponding to the sample’s width dimension.

Vertical (V) direction, corresponding to the sample’s length dimension.

**Figure 4:**
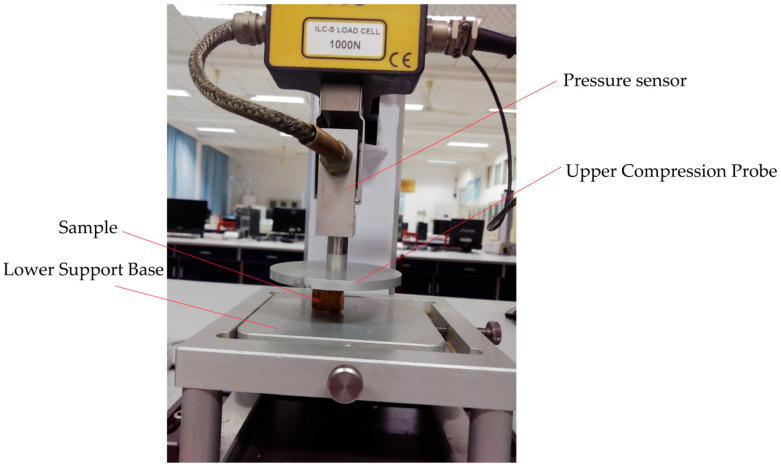
Measurement of texture characteristics (vertical).

This comprehensive multi-directional testing protocol, illustrated schematically in Figure 4, ensured a thorough evaluation of the shell’s viscoelastic properties from all critical orientations.

**Figure 5:**
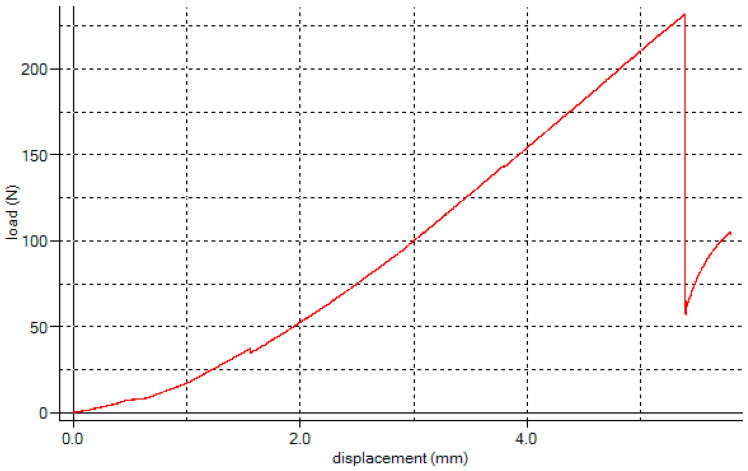
Compression stress–strain curve.

The test sample was placed on the pressure plate, and the sample was compressed at a constant rate of 30 mm/min. When the compression head touched the sample, the sensor collected and recorded the load and displacement data to generate a stress–strain curve as shown in Figure 5.

When the pressure rose to a peak and dropped sharply, it indicated that the sample had been fractured, and the test was over. The elastic modulus of the WCS was the slope of the stress–strain curve of the sample under compression load, and its calculation formula was as follows [30]:

*E* = *FL*/*AΔL* (1)

where *E* is the elastic modulus of the WCS (MPa), *F* is the pressure (N), *A* is the cross-sectional area of the sample (mm^2^), *ΔL* is the compression deformation of the sample in the test process (mm), *L* is the sample length before the test (mm).

The formula of compressive strength was as follows [30]:

*σ_bc_* = *F_max_*/*A* (2)

where *σ_bc_* is the compression strength (MPa), and *F_max_* is the compression limit load (N).

#### 2.3.5. Microstructure Observation

To comprehensively evaluate the structural evolution of WCS under varying storage conditions, scanning electron microscopy (SEM) was employed as the primary technique for direct visualization and analysis of microstructural changes, particularly focusing on pore architecture [31]. Understanding alterations in pore size, distribution, and morphology is critical, as these parameters significantly influence moisture migration, gas exchange, microbial colonization, and overall degradation kinetics. Sample preparation followed a rigorous protocol to ensure imaging fidelity. Representative shell fragments were meticulously sectioned into small, standardized cubes (2 mm × 2 mm × 1 mm) using a sharp blade to minimize structural damage and expose a fresh, representative internal surface for observation. These prepared specimens were then securely mounted onto aluminum SEM stubs using precision tweezers, ensuring stable positioning crucial for high-magnification imaging.

Prior to SEM observation, samples underwent conductive coating to prevent charging artifacts under the electron beam. This was achieved using a high-vacuum sputter coater, where samples were coated with a fine layer of gold-palladium (Au-Pd) for approximately 15 min. The coating process was conducted under a controlled vacuum pressure of 300 Pa with an accelerating voltage of 6 kV, parameters optimized to achieve a uniform, ultra-thin conductive film (~10–20 nm thickness) without obscuring fine surface details.

Microstructural analysis was performed using a Toshiba SU8010 scanning electron microscope [32]. This instrument is renowned for its exceptional resolution at low accelerating voltages. Samples were observed under high vacuum conditions within the microscope chamber at various magnifications (typically ranging from 200× to 500×) to capture both overall surface topography and intricate pore structures. Secondary electron (SE) imaging mode was primarily utilized to generate high-resolution, topographical contrast images, enabling detailed qualitative assessment of surface morphology, pore geometry, crack formation, and any signs of structural collapse or microbial biofilm development resulting from different temperature treatments. Comparative analysis of these micrographs directly revealed the impact of storage temperature on the fundamental pore structure integrity of the WCS.

#### 2.3.6. Statistical Analysis

All experiments were performed in triplicate (n = 3), and the data are presented as the mean ± standard deviation. To determine the significant effects of storage temperature and time on the quality parameters, the data were subjected to one-way or two-way analysis of variance (ANOVA). Specifically, a two-way ANOVA was used to analyze the interactive effects of temperature and time, followed by Dunnett’s post hoc test for comparisons against the control group or between different temperature groups at each time point. Differences were considered statistically significant at a *p*-value of less than 0.05. All statistical analyses were performed using SPSS 26.0 software.

## 3. Results and Discussion

### 3.1. Sensory Quality

Vacuum packing can insulate the sample from air and retain the moisture as much as possible, making it an ideal method to maintain the sensory quality of WCS. However, with the extension of storage time, water immersion may occur in the bag, and the accumulation of gas generated by microorganisms will lead to the bulging of the bag. The rates of water immersion and microbial reproduction are different at different storage temperatures, resulting in different declining rates of sensory quality for the samples.

**Figure 6:**
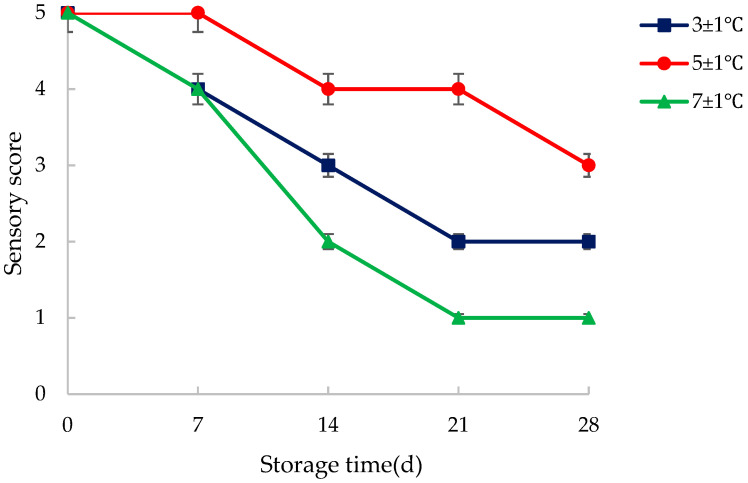
Influence of storage temperature on the sensory quality of vacuum-packed water caltrop shell.

According to the scoring criteria listed in Table A1, samples were taken every seven days to evaluate the sensory quality at three storage temperatures. The results are shown in Figure 6.

As established by the sensory evaluation criteria in Table A1, a sensory score of 4 or higher signifies WCS retaining acceptable to high quality, characterized by minimal deterioration in color, odor, and texture. Conversely, a score of 2 or below indicates significant quality failure, marked by pronounced spoilage characteristics rendering the shell unsuitable for further utilization. Figure 6 clearly demonstrates a universal decline in sensory quality across all tested storage temperatures as time progresses. This downward trajectory is expected but crucial; it confirms that no storage condition tested completely halts degradation, only modulates its rate. The consistent trend underscores the perishable nature of WCS biomass and the relentless impact of biochemical and microbial processes post-harvest, even under controlled temperatures.

**Figure 7:**
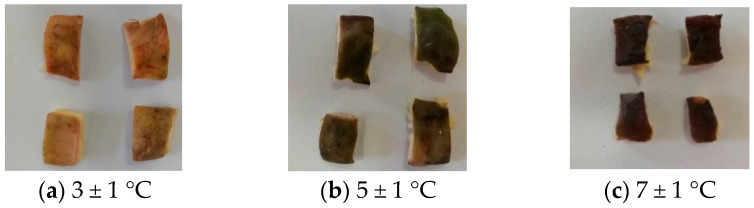
Sensory quality of samples after 14 d of storage.

The dramatic divergence in quality based on storage temperature became starkly evident after 14 days, as visually documented in Figure 7. Comparative analysis of samples revealed profoundly different degradation pathways.

5 ± 1 °C Samples: Exhibited the best preservation. They retained their characteristic green color, indicating minimal chlorophyll degradation or oxidative browning. The texture was only slightly harder, possibly due to controlled moisture loss without cellular collapse. Crucially, no visible mildew was present, suggesting this temperature effectively suppressed the growth of major spoilage fungi and molds. This aligns with the sensory scores likely remaining near or above 4.

3 ± 1 °C Samples: Paradoxically showed inferior preservation compared to 5 °C. A distinct yellowish hue developed, signifying advanced chlorophyll breakdown and potentially the onset of enzymatic browning or other pigment alterations. The texture became slightly soft, implying incipient cellular damage or moisture redistribution issues. Most critically, visible mildew was present, indicating that while some microbial growth was inhibited, psychrotrophic (cold-tolerant) molds were still active. This microbial proliferation directly contributed to the rapid quality decline, likely pushing scores towards 3 or lower.

7 ± 1 °C Samples: Underwent rapid and severe spoilage. The shells turned black, indicative of intense enzymatic browning, non-enzymatic Maillard reactions, and/or prolific microbial growth producing dark pigments. The texture became distinctly soft, signifying extensive cellular disintegration and loss of structural integrity. Heavy mildew confirmed rampant fungal colonization. This combination signifies advanced decay, rendering the shells unusable and correlating with sensory scores plummeting to 2 or below.

This comparative snapshot highlights the extreme sensitivity of WCS quality to minor temperature fluctuations within the refrigerated range. The non-linear relationship is critical: 3 °C was detrimental due to enabling psychrotrophs, while 5 °C struck a balance, slowing metabolic and microbial activity sufficiently for better preservation. 7 °C proved too warm, accelerating all degradation pathways. The presence/absence of mildew is a key differentiator, directly linking moisture content (potentially leading to condensation at colder temps) and temperature-dependent microbial ecology to the observed sensory outcomes and structural collapse.

The experimental results showed that the vacuum-packed samples could maintain a high sensory quality for 21 d when stored at 5 ± 1 °C, while showed a low sensory quality at 21 d and 14 d when stored at 3 ± 1 °C and 7 ± 1 °C, respectively.

The significantly poorer preservation at 3 ± 1 °C compared to 5 ± 1 °C is a key finding, best explained by microbial ecology and physicochemical factors. While 5 ± 1 °C effectively suppresses most spoilage microorganisms, 3 ± 1 °C may selectively favor psychrotrophic molds and bacteria, leading to the observed mildew and quality decline. Additionally, temperatures near 3 ± 1 °C increase the risk of localized freezing or condensation due to minor fluctuations, causing cellular damage that releases nutrients and accelerates spoilage. Thus, 5 ± 1 °C represents an optimal balance, slowing general decay without promoting dominant psychrotrophs or cold-induced damage.

A critical practical implication is the extreme sensitivity of vacuum-packed WCS to a very narrow temperature window. A mere 2 °C deviation from the optimum causes rapid quality failure, highlighting that vacuum packaging is severely limited without precise, unwavering temperature control. This dependency undermines its practicality in real-world supply chains where fluctuations are common.

This limitation defines a crucial direction for future research. Efforts must focus on integrating vacuum packaging with additional hurdles against psychrotrophic microbes, such as natural antimicrobials, tailored modified atmospheres, or surface pre-treatments. Identifying the specific spoilage organisms active at these temperatures is essential for developing robust preservation strategies for this perishable biomass.

### 3.2. Moisture Content

**Figure 8:**
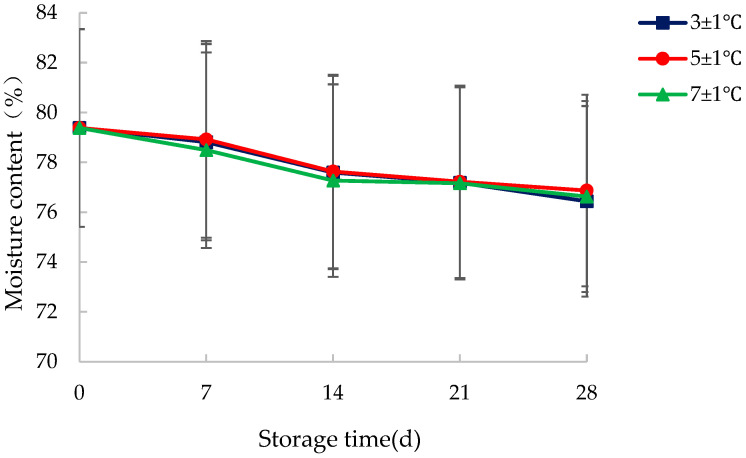
Influence of storage temperature on the moisture content of vacuum-packed water caltrop shell.

Before the storage experiments, the average moisture content of the sample was measured to be 79.38%. Samples were taken in intervals of seven days during the storage to measure the moisture content (Figure 8).

Figure 8 clearly shows that with the extension of storage time, the moisture content of the samples showed a decreasing trend at various storage temperatures. After 28 d of storage, the final average water content of the samples stored at 3 ± 1 °C, 5 ± 1 °C and 7 ± 1 °C was 76.44%, 76.87% and 76.63%, respectively. The decrease in moisture content was no more than 4% for all samples. Moreover, there was no obvious difference in the moisture content of samples among different storage temperatures, indicating that storage temperature has an insignificant effect on the moisture content of vacuum-packed WCS.

Previous studies have shown that agricultural products undergo rapid water loss in a normal temperature environment [33]. Low temperature can significantly reduce the water loss and maintain the freshness of agricultural products [34]. Vacuum packaging can block the contact between the sample and the air to some extent so as to reduce the water loss of the products [35]. Therefore, the fluctuations in the moisture content of samples were generally slight in this experiment.

The observed marginal decrease in moisture content (less than 4%) is a critical finding that strongly validates the efficacy of the combined preservation strategy of vacuum packaging and low-temperature storage. While moisture loss is a primary cause of quality deterioration in fresh agricultural products, the minimal fluctuations recorded here demonstrate that the experimental conditions successfully created a highly stable micro-environment around the samples. This stability is paramount, as it effectively decouples the variable of water loss from other quality parameters measured in the study, allowing for a clearer interpretation of their results. The fact that no significant difference was found among the three storage temperatures further narrows the optimal storage window, suggesting that within this range, temperature is not the dominant factor governing moisture migration. Instead, the vacuum barrier appears to be the primary controller of water vapor transmission. This insight is practically significant for the industry, as it indicates that a slightly higher storage temperature, which may be more energy-efficient, can be adopted without compromising the product’s moisture content, thereby strengthening the paper’s overall conclusion on the robustness of the proposed storage method.

### 3.3. Texture Characteristics

**Figure 9:**
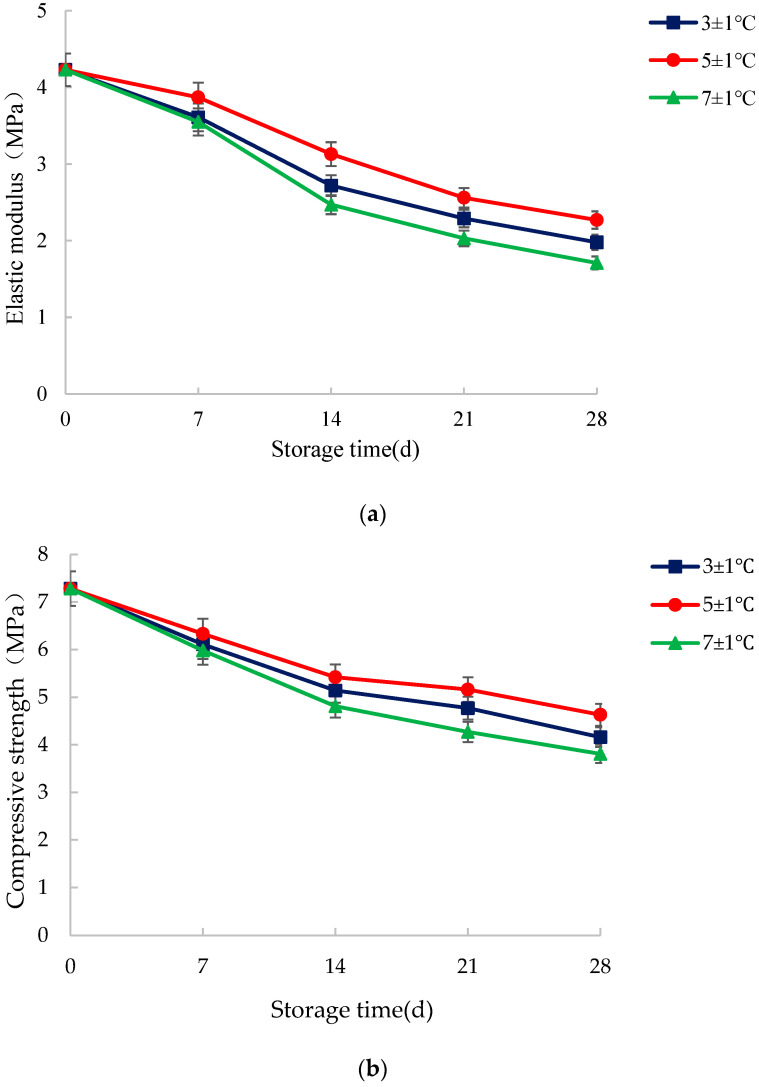
Influence of storage temperature on the elastic modulus (**a**) and compressive strength (**b**) of vacuum-packed water caltrop shell.

In order to prevent the effect of environmental temperature difference at each sampling, the test of texture characteristics was completed within 15 min after sampling. The results are shown in Figure 9.

Before the experiment, the average elastic modulus and compressive strength of the samples were 4.23 MPa and 7.28 MPa, respectively. Figure 9 showed that both elastic modulus and compressive strength gradually decreased with increasing storage time, and the decrease was more significant at the early stage (0–14 d) than at the late stage (14–28 d). In the same storage time, storage at 5 ± 1 °C resulted in the least significant decrease in elastic modulus and compressive strength.

The progressive decline in the mechanical properties of WCS during storage is consistent with the degradation patterns observed in other lignocellulosic biomass. The initial rapid decrease (0–14 d) likely results from the breakdown of soluble components like pectins and hemicelluloses by residual enzymes and early microbial activity. The subsequent slower decline (14–28 d) suggests a shift to the degradation of more resistant polymers such as cellulose and lignin. The superior preservation at 5 ± 1 °C aligns with established principles, as this temperature effectively slows metabolic and microbial processes.

While previous research focused on optimizing shelling mechanisms for fresh produce, our findings demonstrate that the same mechanical integrity crucial for processing is highly vulnerable to post-harvest storage conditions. The non-linear deterioration we observed, particularly the significant impact of minor temperature fluctuations, underscores that mechanical weakening is intrinsically linked to the biochemical and microbial spoilage pathways identified in our sensory analysis. This highlights that temperature control is vital not only for sensory quality but also for maintaining the structural utility of WCS biomass.

### 3.4. Microstructure

**Figure 10:**
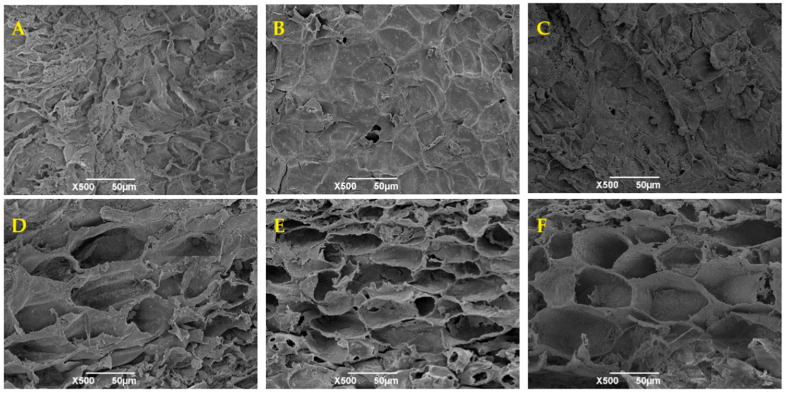
Influence of storage temperature on the microstructure of vacuum-packed water caltrop shell. (**A**) before storage at 3 ± 1 °C; (**B**) before storage at 5 ± 1 °C; (**C**): before storage at 7 ± 1 °C; (**D**) after storage at 3 ± 1 °C; (**E**) after storage at 5 ± 1 °C; (**F**) after storage at 7 ± 1 °C.

Previous studies have shown that irreversible changes will occur in the microscopic pore structure of agricultural products during storage [36,37]. Therefore, to study the effect of storage temperature on the microstructure, it is only necessary to test and compare the samples before and after the storage. To ensure the accuracy of the experimental results, two adjacent sampling sites on the same WCS sample were chosen before and after the storage, and the experimental results are shown in Figure 10.

The results showed that the pore number and pore diameter of the samples increased after 28 d of storage. As shown in Figure 10, the samples had a small pore diameter and compact structure before storage. However, the pore diameter increased, and the microstructure became less compact after storage. Among the three storage temperatures, storage at 7 ± 1 °C led to the most significant change in pore structure, followed by storage at 3 ± 1 °C, while the least significant change was observed in the samples when stored at 5 ± 1 °C.

The observed microstructural changes in WCS—specifically the increase in pore number and diameter and the formation of cavities—align with established literature on the post-harvest deterioration of lignocellulosic materials [36,37]. These alterations are characteristic of cell wall degradation, where the breakdown of structural polymers (e.g., pectins, hemicelluloses) weakens the tissue framework. While SEM directly visualizes the physical manifestations, the primary driver of these changes—microbial growth—is inferred from our physicochemical and sensory data and supported by prior research. The pronounced degradation at 7 °C and the intermediate damage at 3 °C correlate directly with the microbial proliferation (visible mildew) and enzymatic activity recorded in our earlier analyses, confirming that microstructural porosity serves as a physical indicator of underlying biochemical spoilage.

The correlation between microstructural changes and macroscopic quality degradation is direct and consequential. The formation of cavities and cracks represents cell wall collapse and loss of structural integrity, which directly explains the reduction in compressive strength and elastic modulus observed in our mechanical tests. As the cellular framework disintegrates, the material’s ability to resist deformation diminishes. Furthermore, these structural breaches facilitate uncontrolled moisture migration within the tissue, leading to the simultaneous occurrence of localized softening (due to water accumulation in broken cells) and overall texture loss. This breakdown in microstructure provides the physical basis for the sensory quality decline, particularly the soft texture and color changes associated with enzymatic browning and microbial invasion. The superior preservation at 5 °C underscores that inhibiting microstructural decay is fundamental to maintaining both the mechanical and sensory quality of WCS, offering critical insights for optimizing storage conditions to preserve its utility.

The SEM images could directly reveal the changes in the microscopic structure of WCS. Before storage, the samples showed intact cell structure, while after storage, cavities and cracks were observed on the cell wall of WCS. The results showed that storage temperature has a certain effect on the cell wall of WCS and may provide some reference for the development of water caltrop sheller.

### 3.5. Variance Analysis

In order to further explore the effects of storage temperature and storage time on the quality of vacuum-packed WCS, a variance analysis was carried out on the experimental results of sensory quality, moisture content and texture characteristics. The results are shown in Table A2.

The variance analysis results showed that storage time has a significant effect on each test index. The storage temperature has very significant effects on the sensory quality, elastic modulus and compressive strength, but an insignificant effect on the moisture content of the samples. The results also revealed that storage time is the primary factor affecting the storage quality of WCS. Both storage temperature and storage time have significant effects on the texture characteristics of the samples, which are closely related to the microstructure, indicating that storage temperature and storage time also have significant effects on the microstructure of WCS.

The optimal storage temperature and storage time obtained from the experiment may provide reference for the packaging and storage of WCS as well as the development of water caltrop sheller. Future studies may be focused on the effects of packaging materials and methods on the storage quality of WCS.

## 4. Conclusions

This study comprehensively evaluated the impact of storage temperature and time on the quality degradation of WCS. Crucially, storage at 5 ± 1 °C emerged as the optimal condition, demonstrably minimizing detrimental changes across key quality parameters. Samples held at this temperature exhibited significantly superior preservation compared to those at 3 ± 1 °C or 7 ± 1 °C, evidenced by the following:

The least reduction in mechanical integrity, reflected in the smallest decreases in elastic modulus and compressive strength.

The most favorable sensory scores (maintaining acceptability longer) and minimal visible mold growth.

The least severe microstructural degradation, with SEM revealing substantially fewer cavities and cracks in cell walls compared to higher temperatures.

Furthermore, when combined with vacuum packaging, WCS stored at 5 ± 1 °C maintained a high quality standard for up to 21 days, significantly extending the usable shelf-life. Statistical analysis via ANOVA unequivocally identified storage time as the primary factor (*p* < 0.01) driving quality deterioration, exerting a stronger influence than temperature within the tested range. These findings provide critical technical parameters for optimizing the post-harvest management of WCS. They offer a scientifically validated protocol (5 ± 1 °C + vacuum packaging) to reduce waste, preserve bioactive compounds, and ensure raw material consistency, thereby directly supporting the development of value-added food processing technologies and sustainable utilization strategies for this significant agricultural by-product.

While the integrated assessment of sensory, textural, and microstructural parameters provides robust and practical indicators of quality degradation, it is important to acknowledge that these metrics primarily reflect macroscopic and physical manifestations of spoilage. To achieve a more comprehensive and mechanistic understanding of storage suitability, these findings should be complemented in future research with direct chemical and microbiological analyses. Quantifying specific spoilage indicators, such as microbial load, the activity of cell wall-degrading enzymes, and the production of volatile compounds associated with off-odors, would provide definitive evidence for the underlying causes of quality loss. Such an integrated approach would not only validate the physical and sensory observations reported here but also establish critical thresholds for chemical and microbial quality, ultimately leading to more precise and predictive models for WCS shelf-life management.

## Data Availability

The original contributions presented in this study are included in the article. Further inquiries can be directed to the corresponding author.

## Abbreviations

The following abbreviations are used in this manuscript:

WCS	water caltrop shell
SEM	scanning electron microscopy
SE	secondary electron

## Appendix A

**Table A1:** Criteria for sensory quality scoring of water caltrop shell.

Evaluation Index	Evaluation Score				
	5	4	3	2	1
Color	Fresh green	Dark green	Dark yellow	Little black	Black
Odor	Fragrance	No mildew	Slight moldy	Mildew	Heavy mildew
Surface texture	Strong	Little hard	Little soft	Soft	Soft rotten

**Table A2:** Experimental data of water caltrop shell storage.

Factors and Indicators		Sensory Quality	Moisture Content (%)	Elastic Modulus (MPa)	Compressive Strength (MPa)
3 ± 1 °C	0 d	5	79.38	4.23	7.28
	7 d	4	78.92	3.61	6.11
	14 d	3	77.63	2.72	5.14
	21 d	2	77.22	2.29	4.77
	28 d	2	76.87	1.98	4.16
5 ± 1 °C	0 d	5	79.38	4.23	7.28
	7 d	5	78.81	3.87	6.33
	14 d	4	77.59	3.13	5.42
	21 d	4	77.18	2.56	5.16
	28 d	3	76.44	2.27	4.63
7 ± 1 °C	0 d	5	79.38	4.23	7.28
	7 d	4	78.49	3.55	5.98
	14 d	2	77.27	2.47	4.81
	21 d	1	77.16	2.03	4.27
	28 d	1	76.63	1.71	3.81
Effect of storage temperature		F = 9.33 **	F = 3.13	F = 12.07 **	F = 10.46 **
Effect of storage days		F = 14.29 **	F = 201.70 **	F = 148.14 **	F = 131.93 **

Note: ** significant at *p* < 0.01 based on ANOVA.
